# Clinical Characteristics and Surgical Management of Calvarial Tumors: A Single-Center Retrospective Study

**DOI:** 10.3390/jcm15208045

**Published:** 2026-10-16

**Authors:** Onur Boloğur, Hakan Yılmaz, Hüseyin Berk Benek, Erdal Kıssaboylu, Engin Kayıkcı, Serkan Uçmak

**Affiliations:** 1 Department of Neurosurgery, İzmir City Hospital, Bayraklı, 35540 İzmir, Türkiye; 2 Department of Neurosurgery, İzmir Faculty of Medicine, University of Health Sciences, 35100 İzmir, Türkiye; 3 Department of Neurosurgery, Faculty of Medicine, Muğla Sıtkı Koçman University, 48000 Muğla, Türkiye; 4 Department of Endodontics, Faculty of Dentistry, Selçuk University, 42250 Konya, Türkiye

**Keywords:** calvarial tumor, skull neoplasms, osteoma, skull metastasis, surgical resection, cranioplasty, recurrence

## Abstract

**Background/Objectives:** Calvarial tumors form a heterogeneous group of benign and malignant lesions, and institutional series remain limited, particularly from Türkiye. This study aimed to describe the clinical, radiological, histopathological, and surgical features and histology-specific outcomes of calvarial tumors treated at a single neurosurgical center. **Methods:** We retrospectively reviewed 42 patients who underwent surgery for histopathologically confirmed tumors and tumor-like lesions of the cranial vault between 2019 and 2024. Clinical, imaging, histopathological, surgical, and follow-up data were analyzed. **Results:** The cohort comprised 24 men and 18 women (mean age 42.6 ± 15.3 years; range 7–78). A palpable mass was the most common complaint (64%), and the frontal bone was the most common site (38%). Lesions were benign in 25 patients (60%, including 6 with fibrous dysplasia) and malignant in 17 (40%); osteoma and metastasis were the leading diagnoses. Malignant tumors were larger and had a shorter symptom duration than benign lesions. Total resection was achieved in 36 patients (86%). During a mean follow-up of 32 months, recurrence occurred in 8% (95% CI 2–25%) of benign and 35% (95% CI 17–59%) of malignant tumors (*p* = 0.045); 8 recurrences occurred after total resection. **Conclusions:** In this surgical series, a short symptom duration, larger lesion size, and destructive CT features were associated with malignancy, although their diagnostic accuracy requires validation in larger cohorts. Recurrence was uncommon in those with benign lesions but occurred in about one-third of those with malignant tumors, which therefore require multidisciplinary management and close surveillance. Because recurrence also occurred after total resection, the extent of resection alone did not ensure local control.

## 1. Introduction

Calvarial tumors are neoplastic and tumor-like lesions that arise within the bones of the skull vault or involve them secondarily through metastatic spread or direct invasion [1,2,3]. They cover a broad biological spectrum, ranging from slow-growing osteomas and intraosseous hemangiomas to osteosarcomas, Ewing sarcomas, and metastatic carcinomas [2,4]. Because many lesions remain asymptomatic or are managed outside neurosurgical services, their true incidence is unknown, and most available clinical data come from small institutional series and case reports [5,6,7].

The pathogenesis of these tumors differs according to histological type. Osteomas are benign lesions composed of mature bone, whereas primary malignant bone tumors are associated with specific molecular alterations [8]. Most Ewing sarcomas carry the t (11;22) (q24;q12) translocation, which generates the *EWSR1–FLI1* fusion gene [9], and *IDH1* or *IDH2* mutations are common in central chondrosarcomas [10]. Secondary involvement of the skull most commonly originates from breast, lung, prostate, thyroid, and renal carcinomas [11,12].

Clinical presentation varies with histology and location. Benign lesions usually present as a painless, slowly enlarging mass or a cosmetic deformity, whereas malignant tumors more often cause pain, rapid growth, and neurological symptoms when they extend intracranially [2,5]. Cross-sectional imaging is central to preoperative planning: computed tomography (CT) best shows the pattern of bone destruction, matrix mineralization, and cortical involvement, while magnetic resonance imaging (MRI) depicts marrow replacement, soft-tissue extension, dural involvement, and intracranial spread [1,4,13]. However, imaging features often overlap, and a definitive diagnosis requires histopathological examination [5,7].

Treatment is determined largely by histology. Complete excision is curative for most benign lesions, and the resulting defect can be reconstructed in the same session [5,6]. For primary malignant tumors and metastases, surgery is usually one component of a multimodal strategy that may include radiotherapy, chemotherapy, or radiosurgery, tailored to tumor type and systemic disease status [11,14,15]. Several institutional series of calvarial lesions have been published; however, the largest recent series originate from Western European centers [5,7], and data from Türkiye are limited [6]. Moreover, most series compare benign and malignant lesions as two groups, and adjuvant treatment, recurrence according to histology and extent of resection, and the complication profile of resection with cranioplasty are often not reported in detail. The aim of this study was to describe the clinical presentation, imaging findings, histopathological spectrum, surgical management, and outcomes of patients operated on for calvarial tumors at a tertiary neurosurgical center in Türkiye, to compare benign and malignant lesions, and to report treatment and recurrence according to histological subtype and extent of resection.

## 2. Materials and Methods

### 2.1. Study Design and Patient Selection

This retrospective cohort study included all patients who underwent surgery for a calvarial tumor at the Department of Neurosurgery, Izmir City Hospital, between 2019 and 2024 and who met the eligibility criteria. Calvarial lesions were defined as lesions arising from, or primarily involving, the bones of the cranial vault (frontal, parietal, squamous temporal, and squamous occipital bones); lesions confined to the skull base, including the orbital roof, sphenoid wing, and petrous temporal bone, were not included. Inclusion criteria were a definitive histopathological diagnosis of a calvarial tumor or tumor-like lesion and complete preoperative CT and MRI. Patients with bone lesions of traumatic or infectious origin and those with suspected calvarial tumors without an available histopathological diagnosis were excluded. During the study period, 62 patients underwent surgery for a calvarial lesion; 11 were excluded because of a traumatic or infectious origin, absence of a definitive histopathological diagnosis (*n* = 5), or incomplete preoperative imaging (*n* = 4), leaving 42 patients for analysis. Multifocal calvarial involvement was present in 2 patients; these patients were counted once and classified according to the surgically treated lesion. Because only surgically treated, histologically confirmed lesions with complete preoperative imaging were included, the cohort represents a selected surgical population rather than the full spectrum of calvarial tumors. The study protocol was approved by the Non-Interventional Clinical Research Ethics Committee of Izmir City Hospital (decision No. 2026/33, 4 February 2026).

### 2.2. Data Collection and Definitions

Clinical records, the radiological archive, and operative notes were reviewed for each patient. The following variables were recorded: age, sex, presenting symptoms and symptom duration, tumor location and size, imaging characteristics, histopathological diagnosis, extent of resection, method of cranial reconstruction, postoperative complications, adjuvant treatment (type and timing), recurrence (time to recurrence and its management), and length of follow-up. Lesions were classified as benign or malignant according to the final histopathological diagnosis. Fibrous dysplasia, a non-neoplastic tumor-like lesion, was included in the benign group because its clinical presentation, differential diagnosis, and surgical management overlap with those of calvarial tumors; because of its non-neoplastic nature, all benign–malignant comparisons were repeated after excluding patients with fibrous dysplasia (sensitivity analysis). Tumor size was defined as the maximum lesion diameter measured on preoperative imaging, and recurrence was defined as new or enlarging tumor at the operative site on follow-up CT or MRI.

### 2.3. Imaging Evaluation

All patients underwent CT and MRI before surgery. CT was used to assess bone architecture, lesion margins, cortical destruction, and periosteal reaction, and MRI was used to evaluate soft-tissue, dural, and intracranial extension. All images were reviewed to assess sclerotic or lytic patterns, margin definition (well-defined or irregular), cortical destruction, periosteal reaction, and soft-tissue and intracranial extension. When malignancy was suspected, positron emission tomography–computed tomography (PET-CT) was performed for systemic staging. The definitive diagnosis was established in all cases by histopathological examination of surgical or tru-cut biopsy specimens.

### 2.4. Surgical Management and Adjuvant Treatment

The extent of resection (total or subtotal) was planned according to lesion location and size, the relationship of the tumor to adjacent vascular and neural structures, and the suspected histology. Total resection was defined as complete removal of the lesion, including the involved bone, with no residual tumor on postoperative CT and/or MRI obtained during or after surgery; subtotal resection was defined as any residual tumor visible on postoperative imaging. The surgeon’s intraoperative assessment was recorded in the operative notes, and pathological margin status was available for patients. Skull defects created during surgery were reconstructed with autologous bone grafts or titanium mesh. For patients with a malignant histopathological diagnosis, the need for adjuvant therapy was determined by the institutional multidisciplinary tumor board. Follow-up consisted of clinical examination and CT or MRI at 1 year.

### 2.5. Statistical Analysis

Statistical analyses were performed using IBM SPSS Statistics software (version 25; IBM Corp., Armonk, NY, USA). Categorical variables are presented as numbers and percentages, and 95% confidence intervals (CIs) for key proportions were calculated with the Wilson score method. The distribution of continuous variables was assessed with the Shapiro–Wilk test and visual inspection of histograms. Normally distributed variables are presented as mean ± standard deviation (SD) and were compared with Student’s *t*-test; non-normally distributed variables, including symptom duration, are presented as median (interquartile range, IQR) and were compared with the Mann–Whitney U test. Categorical variables were compared using the chi-square test or, when any expected cell count was below five, Fisher’s exact test. Exact *p* values are reported, except when *p* < 0.001. Because of the small sample size and the low number of events, no multivariable analysis or diagnostic performance analysis (receiver operating characteristic curves, cut-off values, sensitivity, or specificity) was performed. The analyses were exploratory, and no adjustment for multiple comparisons was made; *p* values should therefore be interpreted with caution. A two-sided *p* value < 0.05 was considered statistically significant.

## 3. Results

### 3.1. Demographic and Clinical Characteristics

Of the 42 patients, 24 (57%) were male and 18 (43%) were female, with a mean age of 42.6 ± 15.3 years (range, 7–78 years; median, 42 years). The age distribution was bimodal: 12 patients (29%) were aged 20–30 years and 13 (31%) were older than 50 years. Five patients were younger than 18 years (age range, 1–16 years); their histological diagnoses were hemangioma, osteoma and fibrous dysplasia. Malignant tumors were more common in male than in female patients (*p* = 0.041).

The most common presenting complaint was a palpable mass (*n* = 27, [64%]), followed by headache (*n* = 16, [38%]), cosmetic deformity (*n* = 11, [26%]), and local tenderness (*n* = 9, [21%]) (Table 1). Neurological deficits were present in 3 patients (7%), all of whom had malignant tumors. Patients with malignant tumors more often presented with multiple complaints, and symptom duration before diagnosis was shorter in the malignant group than in the benign group (median 8.1 vs. 11.1 months; mean 6.8 vs. 14.2 months; *p* = 0.01, Mann–Whitney U test).

### 3.2. Tumor Location and Histopathological Findings

The frontal bone was the most common location (*n* = 16, 38%), followed by the parietal (*n* = 12, 29%), temporal (*n* = 8, 19%), and occipital (*n* = 6, 14%) bones. Histopathological examination identified benign lesions in 25 patients (60%) and malignant lesions in 17 (40%) (Table 2). Osteoma was the most common benign diagnosis (*n* = 12, 29% of the entire cohort), followed by hemangioma (*n* = 7, 17%) and fibrous dysplasia (*n* = 6, 14%). Metastases formed the largest malignant group (*n* = 10, 24%), followed by osteosarcoma (*n* = 4, 10%) and Ewing sarcoma (*n* = 3, 7%). The primary sites of the metastatic tumors were the breast (*n* = 4), lung (*n* = 3), thyroid (*n* = 2), and prostate (*n* = 1). The sensitivity analysis excluded the 6 patients with fibrous dysplasia (19 benign tumors vs. 17 malignant tumors).

### 3.3. Radiological Findings

Malignant tumors were significantly larger than benign lesions (mean size 4.1 vs. 2.3 cm; median 5.1 vs. 3.1 cm; *p* < 0.001). On CT, benign lesions were typically sclerotic and well circumscribed, whereas malignant lesions more frequently showed cortical destruction, irregular margins, and periosteal reaction (Table 3). MRI demonstrated soft-tissue extension in 9 patients (21%) and intracranial extension in 6 patients (14%), 5 of whom had malignant tumors (*p* = 0.032).

### 3.4. Surgical Treatment, Complications, and Adjuvant Therapy

Total resection was achieved in 36 patients (86%; 95% CI 72–93%), and subtotal resection was performed in 6 patients (14%). Subtotal resection was preferred when the tumor was closely related to critical vascular or neural structures or showed extensive bone infiltration.

No perioperative mortality occurred. Postoperative complications developed in 6 patients (14%) (Table 4). Three patients required reoperation for evacuation of a postoperative hematoma at the surgical site (epidural, *n* = 2; subdural, *n* = 1). Two patients underwent wound revision for cerebrospinal fluid (CSF) leakage and were discharged without further complications. One patient developed a decreased level of consciousness; further investigation revealed an ischemic event, and the patient was discharged with paresis after medical treatment.

In the malignant group, 14 of 17 patients (82%) received postoperative radiotherapy and/or chemotherapy. Among the 10 patients with metastases, 8 received radiotherapy to the resection site (conventional radiotherapy, *n* = 8; stereotactic radiosurgery, *n* = 1) and 2 received systemic therapy for the primary tumor. Of the 4 patients with osteosarcoma, 2 received chemotherapy and 2 received radiotherapy; of the 3 patients with Ewing sarcoma, 2 received chemotherapy and 1 received radiotherapy. The 3 patients with malignant tumors who did not receive adjuvant treatment had poor Karnofsky scores. Treatment and recurrence according to histological subtype are summarized in Table 5. Adjuvant treatment decisions were based on histological type, surgical margin status, and metastatic potential.

### 3.5. Follow-Up and Recurrence

**Figure 1:**
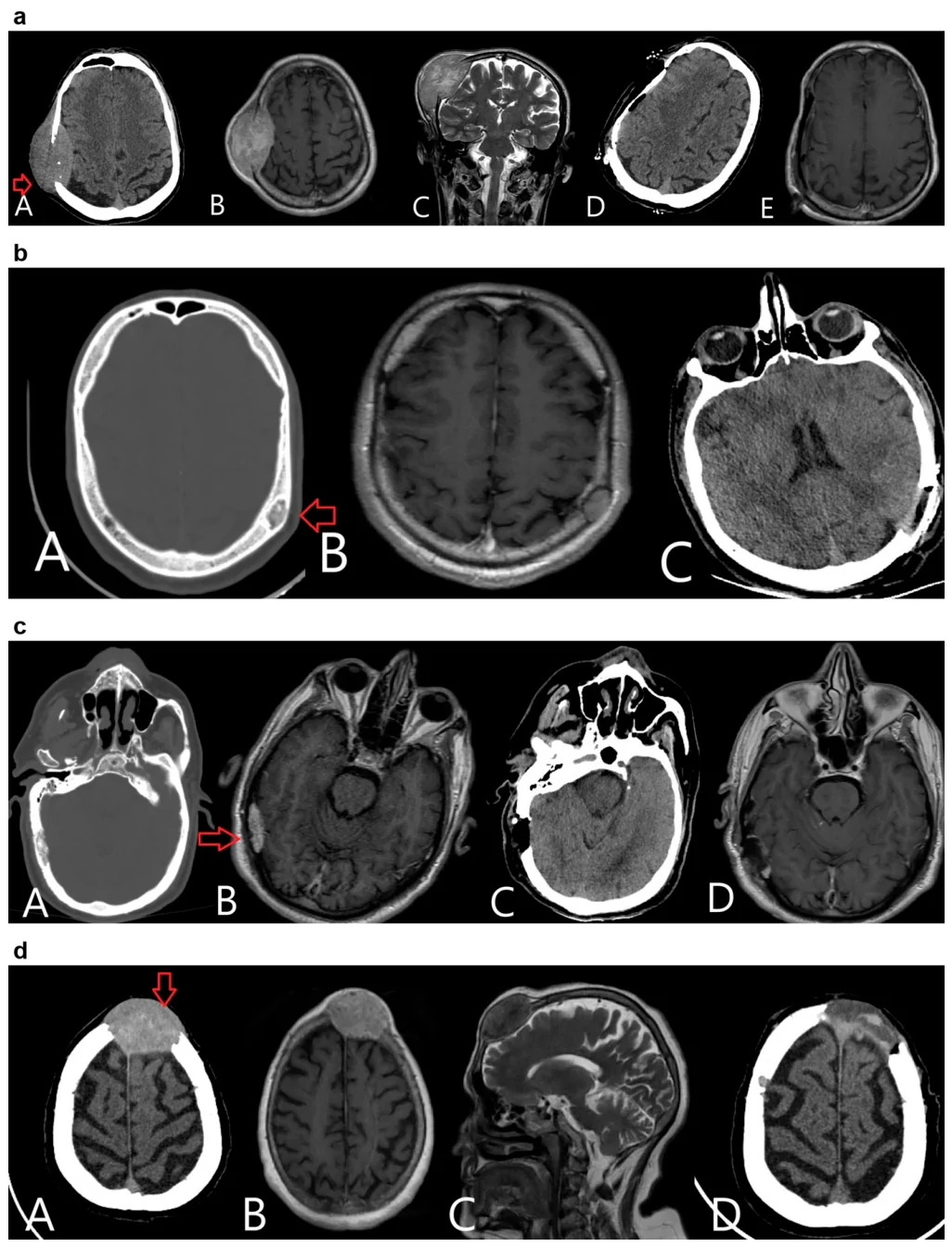
Representative preoperative and postoperative images. (**a**) Calvarial metastasis of carcinoma with dural involvement: (**A**) axial CT, (**B**) axial MRI, and (**C**) coronal MRI obtained preoperatively; (**D**) postoperative axial CT and (**E**) postoperative axial MRI. (**b**) Calvarial osteoma: (**A**) axial CT and (**B**) axial MRI obtained preoperatively; (**C**) postoperative axial CT. (**c**) Calvarial hemangioma: (**A**) axial CT and (**B**) axial MRI obtained preoperatively; (**C**) postoperative axial CT and (**D**) postoperative axial MRI. (**d**) Calvarial osteosarcoma with dural involvement: (**A**) axial CT, (**B**) axial MRI, and (**C**) sagittal MRI obtained preoperatively; (**D**) postoperative axial CT. Red arrows indicate the tumors.

The mean follow-up period was 32 months (range, 6–60 months; median, 36 months). Recurrence was observed in 8 patients (19%; 95% CI 10–33%): 2 of 25 patients with benign lesions (8%; 95% CI 2–25%) and 6 of 17 patients with malignant tumors (35%; 95% CI 17–59%) (*p* = 0.045, Fisher’s exact test). Recurrence occurred in 2 of 36 patients after total resection and in 4 of 6 patients after subtotal resection (*p* = 0.001, Fisher’s exact test); because only 6 patients underwent subtotal resection, at least 2 of the 8 recurrences occurred after total resection. The histology, extent of resection, adjuvant treatment, and time to recurrence of each patient with recurrence are shown in Table 6. Given the small number of events, the association between extent of resection and recurrence could not be assessed independently of histology. Representative preoperative and postoperative images are shown in Figure 1.

## 4. Discussion

In this single-center series of 42 surgically treated calvarial lesions, benign lesions predominated, with osteoma being the most common diagnosis, whereas metastases accounted for most malignant tumors. Compared with benign lesions, malignant tumors were associated with a shorter symptom duration, larger size, destructive CT features, intracranial extension, and a higher recurrence rate in univariable comparisons. These observations are broadly consistent with previous institutional series and radiological reviews [5,6,7]. Our series adds contemporary data from a Turkish tertiary center in which all patients had complete preoperative CT and MRI, together with a detailed complication profile and patient-level treatment and recurrence data according to histology and extent of resection.

The bimodal age distribution in our cohort reflects the different age preferences of the underlying pathologies: benign bone lesions are commonly diagnosed in younger and middle-aged adults, whereas metastatic disease becomes more common with advancing age [1,2]. As in the Tübingen series, in which swelling, pain, cosmetic concerns, and neurological deficits were the main complaints [5], a palpable mass was the leading reason for presentation in our patients. Notably, all neurological deficits occurred in the malignant group, and their symptom duration was roughly half that of the benign lesion group. Clinically, a painful, rapidly enlarging lesion or one accompanied by neurological signs should therefore prompt an expedited work-up for malignancy, including systemic staging.

The frontal and parietal bones were the most commonly affected sites, in line with earlier reports [5,6]. This distribution may partly reflect the larger surface area of these bones rather than a specific biological predisposition.

The histological spectrum of our series differed in some respects from those of larger European cohorts. Nasi-Kordhishti et al. reported histiocytosis, meningioma, metastasis, and osteoma as the most common diagnoses among 133 patients [5], and Harapan et al. found meningiomas and metastases to be the most common entities among 174 patients, 66.1% of whom had benign lesions [7]. No intraosseous meningioma or Langerhans cell histiocytosis was identified in our cohort, which may be related to referral patterns, the relatively short study period, and the fact that pediatric lesions are often treated in dedicated pediatric centers. Fibrous dysplasia is a tumor-like lesion rather than a true neoplasm; it was included because its clinical presentation, differential diagnosis, and surgical management overlap with those of calvarial tumors [2,3], and a sensitivity analysis excluding these patients is reported in Section 3.2.

Imaging remains the cornerstone of preoperative assessment. Well-defined sclerotic margins favor a benign process, whereas permeative destruction, irregular borders, periosteal reaction, and soft-tissue or intracranial extension suggest malignancy [1,3,4,13]. In a recent series, multiple osteolytic lesions on CT emerged as the strongest independent predictor of malignancy [7]. In our cohort, malignant tumors were also significantly larger, suggesting that lesion size may provide additional information during preoperative risk assessment. However, because no diagnostic performance analysis or multivariable model was performed, these features should be regarded as associations rather than validated predictors, and their sensitivity, specificity, and optimal cut-off values need to be established in larger cohorts. Nevertheless, imaging patterns overlap considerably, and histopathological confirmation remains mandatory before definitive oncological treatment is planned [5].

The rate of total resection in our study (86%) is comparable with the gross total resection rate of 82.8% reported by Harapan et al. [7]. Recurrence was uncommon in those with benign lesions but occurred in more than one-third of those with malignant tumors. Recurrence was not confined to patients who underwent subtotal resection: at least 2 of the 8 recurrences occurred after total resection, which suggests that histology and systemic disease status, and not only the extent of resection, may influence local control. Because of the small number of events, we could not determine whether residual tumor was an independent risk factor for recurrence in our series. In the literature, residual tumor is a recognized risk factor for regrowth; even a histologically benign calvarial hemangioma has been reported to recur aggressively and invade the brain after repeated subtotal resections [16]. Accordingly, en bloc excision with a margin of normal bone followed by immediate cranioplasty should be pursued whenever it can be performed safely [5,6]. Most complications in our series were related to postoperative hematoma or CSF leakage, underscoring the importance of meticulous epidural hemostasis and watertight dural closure when the dura is opened or resected.

Metastases were the most common malignant tumors, with breast and lung carcinomas being the leading primary sources, which is consistent with previous reports [11,12]. Calvarial metastases may present in varied ways, occasionally as the first manifestation of an unknown primary malignancy [17]. Surgery provides tissue diagnosis, decompression, and local control, but it should be integrated into a multimodal treatment plan. Zhou et al. observed in situ recurrence in 3 of 12 patients after resection of calvarial metastases and recommended wider resection combined with local radiotherapy [14]; stereotactic radiosurgery is an effective alternative for selected patients who are not surgical candidates [15]. The high recurrence rate in the malignant group in our study supports close radiological surveillance and multidisciplinary follow-up for these patients. Because metastases, osteosarcomas, and Ewing sarcomas differ substantially in prognosis, surgical indication, and adjuvant treatment, the pooled recurrence rate of the malignant group should be interpreted together with the histology-specific data presented in Table 5 and Table 6.

This study has several limitations. First, its retrospective single-center design is subject to selection and information bias. Only surgically treated patients with histological confirmation and complete CT and MRI were included; lesions managed conservatively, followed radiologically, or treated with radiotherapy or systemic therapy alone were not captured. Our cohort therefore represents a selected surgical population, and our findings, including the distribution of histological types and the recurrence rates, cannot be generalized to all calvarial tumors. Second, the sample size, particularly within individual histological subgroups, was small, and both the benign and the malignant groups were heterogeneous, which limited statistical power and precluded multivariable analysis and diagnostic performance analysis of factors associated with malignancy or recurrence. In addition, multiple univariable comparisons were performed without adjustment for multiplicity, and these results should be considered exploratory. Third, the follow-up period varied widely (6–60 months) and may have been insufficient to capture late recurrences, especially in slow-growing benign lesions. Finally, although recurrence is reported according to histology and extent of resection, the number of events was too small for formal subgroup analysis, and overall survival and functional outcomes were not evaluated. Larger multicenter studies with standardized imaging assessment and longer follow-up are needed to validate these findings.

## 5. Conclusions

Calvarial tumors encompass a wide range of benign and malignant pathologies, and their management requires a multidisciplinary approach. In our surgical series, a short symptom duration, neurological deficits, larger lesion size, and destructive CT features were associated with malignancy; however, their diagnostic value requires validation in larger studies. Recurrence was uncommon with benign lesions, whereas malignant tumors, most of which were metastases, showed a higher recurrence rate and therefore warrant close follow-up. Because recurrence also occurred after total resection, the extent of resection alone should not be regarded as a guarantee of local control, although total resection should still be pursued whenever it can be performed safely.

## Figures and Tables

**Table 1:** Presenting complaints of the patients (*n* = 42).

Complaint	*n*	%
Palpable mass	27	64
Headache	16	38
Cosmetic deformity	11	26
Local tenderness	9	21
Neurological deficit	3	7

Some patients had more than one complaint; therefore, percentages do not add up to 100%.

**Table 2:** Histopathological distribution of calvarial tumors (*n* = 42).

Histopathological Diagnosis	*n*	% of Cohort
Benign lesions	25	60
Osteoma	12	29
Hemangioma	7	17
Fibrous dysplasia	6	14
Malignant lesions	17	40
Metastasis	10	24
Breast carcinoma	4	10
Lung carcinoma	3	7
Thyroid carcinoma	2	5
Prostate carcinoma	1	2
Osteosarcoma	4	10
Ewing sarcoma	3	7

**Table 3:** CT and MRI findings for benign and malignant lesions (*n* = 42).

Finding	Benign (*n* = 25), *n* (%)	Malignant (*n* = 17), *n* (%)	*p*
CT			
Sclerotic pattern	[22]	[5]	[0.06]
Lytic pattern	[5]	[13]	[0.01]
Well-defined margins	[23]	[11]	[0.03]
Irregular margins	[2]	[6]	[0.05]
Cortical destruction	[18]	[14]	[0.07]
Periosteal reaction	[16]	[10]	[0.04]
MRI			
Soft-tissue extension	[18]	[9]	[0.09]
Intracranial extension	1 (4)	5 (29)	0.032
Size, cm			
Mean ± SD	2.3	4.1	<0.001
Median (IQR)	[3.1]	[5.1]	

Categorical variables were compared with the chi-square test or Fisher’s exact test, as appropriate; size was compared based on QR, interquartile range. SD, standard deviation.

**Table 4:** Postoperative complications (*n* = 42).

Complication	*n*	%	Management
Epidural hematoma	2	5	Surgical evacuation
Subdural hematoma	1	2	Surgical evacuation
CSF leakage from the wound	2	5	Wound revision
Ischemic event	1	2	Medical treatment
Total	6	14	—

CSF, cerebrospinal fluid. No perioperative mortality was observed.

**Table 5:** Treatment and recurrence according to histological subtype (*n* = 42).

Histology	*n*	Total Resection, *n*	Subtotal Resection, *n*	Radiotherapy, *n*	Systemic Therapy, *n*	Recurrence, *n* (%)
Benign lesions	25	[23]	[2]	[0]	[0]	2 (8)
Osteoma	12	[12]	[0]	[0]	[0]	[0]
Hemangioma	7	[7]	[0]	[0]	[0]	[0]
Fibrous dysplasia	6	[4]	[2]	[0]	[0]	[2]
Malignant tumors	17	[13]	[4]	[11]	[6]	6 (35)
Metastasis	10	[9]	[1]	[8]	[2]	[2]
Osteosarcoma	4	[3]	[1]	[2]	[2]	[2]
Ewing sarcoma	3	[1]	[2]	[1]	[2]	[2]
Total	42	36	6	[11]	[6]	8 (19)

Fourteen of the 17 patients with malignant tumors received radiotherapy and/or systemic therapy. Systemic therapy includes chemotherapy, hormonal therapy, and targeted therapy. Patients who received both radiotherapy and systemic therapy are counted in both columns.

**Table 6:** Characteristics of the patients with recurrence (*n* = 8).

Patient	Age/Sex	Histology	Extent of Resection	Adjuvant Treatment	Time to Recurrence, Months	Management of Recurrence
1	[38]	[osteosarcoma]	[Subtotal]	[Radiotherapy]	[24 months]	[reoperation]
2	[13]	[Fibrous dysplasia]	[Total]	[-]	[30 months]	[reoperation]
3	[47]	[Metastasis]	[Total]	[Radiotherapy]	[12 months]	[reoperation]
4	[71]	[Ewing sarcoma]	[Subtotal]	[Systemic therapy]	[6 months]	[Systemic therapy]
5	[68]	[Ewing sarcoma]	[Total]	[Radiotherapy]	[3 months]	[reoperation]
6	[39]	[Fibrous dysplasia]	[Subtotal]	[Systemic therapy]	[24 months]	[reoperation]
7	[82]	[Metastasis]	[Total]	[Radiotherapy]	[16 months]	[Systemic therapy]
8	[76]	[osteosarcoma]	[Subtotal]	[Systemic therapy	[8 months]	[Systemic therapy]

Two recurrences occurred with benign lesions and 6 occurred with malignant tumors.

## Data Availability

The data presented in this study are available on reasonable request from the corresponding author. The data are not publicly available because of patient privacy restrictions.

## Abbreviations

The following abbreviations are used in this manuscript:

CI	Confidence interval
CSF	Cerebrospinal fluid
CT	Computed tomography
IQR	Interquartile range
MRI	Magnetic resonance imaging
PET-CT	Positron emission tomography–computed tomography
SD	Standard deviation
